# Expression of VEGF, EGF, and Their Receptors in Squamous Esophageal Mucosa, with Correlations to Histological Findings and Endoscopic Minimal Changes, in Patients with Different GERD Phenotypes

**DOI:** 10.3390/ijerph19095298

**Published:** 2022-04-27

**Authors:** Justyna Wasielica-Berger, Paweł Rogalski, Agnieszka Świdnicka-Siergiejko, Anna Pryczynicz, Joanna Kiśluk, Jarosław Daniluk, Stefania Antonowicz, Dominik Maślach, Michalina Krzyżak, Andrzej Dąbrowski

**Affiliations:** 1 Department of Gastroenterology and Internal Medicine, Medical University of Bialystok, 15-276 Bialystok, Poland; progalsky@gmail.com (P.R.); agnieszka.swidnicka-siergiejko@umb.edu.pl (A.Ś.-S.); jaroslaw.daniluk@umb.edu.pl (J.D.); antonowiczstefania@gmail.com (S.A.); adabrows@umb.edu.pl (A.D.); 2 Department of General Pathomorphology, Medical University of Bialystok, 15-269 Bialystok, Poland; anna.pryczynicz@umb.edu.pl; 3 Department of Clinical Molecular Biology, Medical University of Bialystok, 15-269 Bialystok, Poland; jk18@interia.pl; 4 Department of Public Health, Medical University of Bialystok, 15-295 Bialystok, Poland; dominikm@umb.edu.pl; 5 Department of Hygiene, Epidemiology and Ergonomics, Medical University of Bialystok, 15-022 Bialystok, Poland; michalina.krzyzak@umb.edu.pl

**Keywords:** VEGF, EGF, growth factors, GERD, minimal change esophagitis

## Abstract

Background: Gastroesophageal reflux disease (GERD) may present as nonerosive reflux disease (NERD), erosive esophagitis (EE), or be complicated by Barrett’s esophagus (BE). The explanation as to what determines the phenotype of GERD is awaited. Therefore, we assessed the correlation between the growth factors expression and endoscopic as histologic findings in GERD patients. Methods: The squamous esophageal epithelium of 50 patients (20-NERD, 7-EE, 15-BE, 8 controls) was examined by: (1) magnification endoscopy with evaluation of minimal GERD changes such as: microerosions, white spots, palisade blood vessels visibility, and intrapapillary capillary loops (IPCLs) appearance, (2) histology, (3) immunohistochemistry with evaluation of the expression of vascular endothelial growth factor (VEGF), epidermal growth factor (EGF), and their receptors (VEGFR and EGFR). Results: The expression of VEGF, but not VEGFR, EGF, and EGFR, was significantly increased in EE patients compared to NERD patients and controls. VEGF levels correlated significantly with the presence of white spots, but not with other minimal endoscopic and histologic features. The EGFR expression correlated positively with basal cell hyperplasia and enlarged IPCLs. Conclusions: Our findings suggest a correlation between growth factors expression and findings in conventional endoscopy, formation of endoscopic minimal changes, and histologic lesions.

## 1. Introduction

Gastroesophageal reflux disease (GERD) is one of the most common gastroenterological problems, and occurs in 20–40% of the population in developed countries [1,2]. In Asia the prevalence of GERD has increased from 11.0% to 15.0% in just the last 20 years [3]. GERD is the most frequent diagnosis in gastroenterological outpatient clinics, and presents a great burden to health resources and adversely impacts health-related quality of life [4]. GERD also continues to be a common indication for upper gastrointestinal endoscopy, reaching 23.9% of all procedures in the United States [4].

In most GERD patients, conventional endoscopy does not detect mucosal breaks in the esophagus. This group is classified as nonerosive reflux disease (NERD). In other patients GERD may proceed with esophageal injury endoscopically visible as erosions or ulcers (erosive esophagitis—EE) or may be complicated with Barrett’s esophagus (BE), which is diagnosed in the presence of columnar or intestinal metaplasia in the esophagus [5,6,7]. EE may lead to serious complications such as gastrointestinal bleeding or esophageal stenosis. On the other hand, BE is among the strongest known risk factors for esophageal adenocarcinoma, incidence of which has risen 6-fold over the last 40 years [8,9]. Esophageal cancer is the sixth most common cause of cancer-related deaths, with a 5-year survival rate of <20.0% despite advances in treatment [3]. The symptoms’ severity and pH-metric values are poor predictors of esophageal mucosal damage [1,10,11]. Therefore, so far there is no simple explanation as to why some patients develop erosive esophagitis or Barrett’s esophagus and others do not.

In addition to the lesions detected during standard endoscopy, such as erosions, ulcers, stenosis, or Barrett’s metaplasia, advanced endoscopic techniques like magnification endoscopy, i-Scan, or NBI endoscopy can detect so-called minimal changes in GERD patients: apical mucosal breaks (microerosions), triangular indentations of Z line, villous mucosa or elongated pit pattern distal to Z line, loss of the visibility of the palisade vessels, punctate erythema, pinpoint blood vessels which represent elongated and/or widened intrapapillary capillary loops (IPCLs), and white points encircling or replacing IPCLs [12,13,14,15]. The last three types of features listed above concern vessels of the squamous mucosa. Minimal endoscopic changes were found in all phenotypes of GERD with the median number of feature types equaling 5 per patient in EE or BE group and 2 per patient in NERD group [13]. Although the samples for histological examination are not taken routinely in GERD, they are helpful in discerning reflux esophagitis from esophagitis of other etiologies [16]. The typical histological changes found in GERD include elongation of the mucosal papillae exceeding 2/3 of epithelial thickness, basal cell hyperplasia exceeding 15% of epithelial thickness, inflammatory cells infiltration (more than 10 leukocytes per high power field), dilated intercellular spaces (DIS), and erosions [5,14,17].

Formation of histologic and minimal endoscopic changes may be a result of repair and healing processes in response to epithelial damage caused by reflux. However, the pathogenesis of individual lesion types is still not sufficiently explained. Notably, the data on the expression and influence of growth factors on the histological features of GERD are scant and the correlation between minimal endoscopic changes and growth factors has not been studied.

Vascular endothelial growth factor (VEGF), which is a strong positive regulator of angiogenesis, plays a key role in repair processes [18]. VEGF stimulates proliferation, migration, differentiation, and cell survival necessary to the formation of new vessels. It has also been identified as a strong vessel permeability factor, allowing circulating inflammatory cells to migrate from the bloodstream into the tissue. An increase in number and size of IPCLs visible at magnifying endoscopy in GERD patients so as the elongation of the papillae containing vessels in histopathology may reflect increased density of blood vessels, which is commonly observed during proliferative phase of healing [19]. This is the reason that we have hypothesized that VEGF plays a role in formation of minimal endoscopic and pathologic lesions.

A previous study using real-time polymerase chain reaction (PCR) showed in 40 EE patients that VEGF mRNA expression was higher in injured mucosa compared to normal mucosa above the injuries [20]. Taddei et al. proved by western blotting of the samples from 32 patients that expression of VEGF in the mucosa above gastro-esophageal junction increased progressively from healthy controls through NERD to EE and BE patients [21]. In contrast, another study on 25 patients did not find significant correlation between VEGF mRNA expression measured by reverse transcription PCR and the severity of reflux esophagitis [22].

Epidermal growth factor (EGF) is another essential cytokine in wound healing and epidermal regeneration [23]. It has been found that luminal release of EGF was significantly lower in patients with EE compared with controls during perfusion with HCl and this decrease was considered as one of the potential mechanisms leading to damage of esophageal mucosa during reflux episodes [24]. However, the data on the EGF expression in GERD patients are inconsistent [20,25]. In addition, the VEGF and EGF expression in patients with minimal endoscopic changes has not yet been explored.

The aim of our study was to analyze the expression of VEGF and EGF and their receptors (VEGFR and EGFR) in the squamous esophageal mucosa in relation to: (1) GERD phenotype, (2) presence of particular types of minimal endoscopic changes, and (3) histologic abnormalities characteristic of GERD.

## 2. Materials and Methods

**Figure 1:**
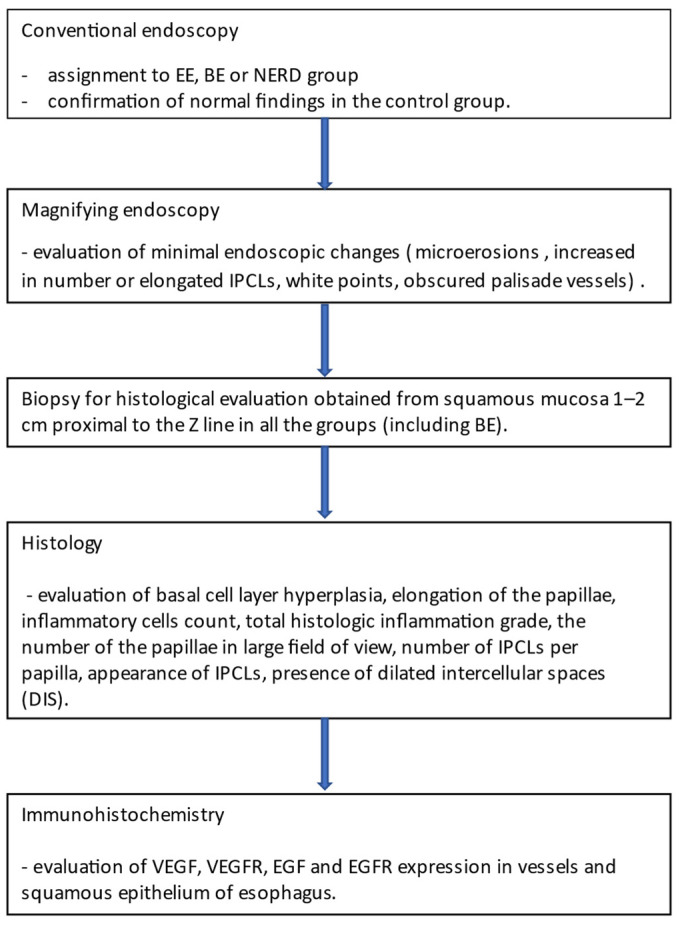
Study design.

We enrolled prospective patients diagnosed with GERD based on the presence of typical reflux symptoms and positive response to proton pump inhibitor (PPI) treatment as well as patients with previously diagnosed BE who were referred for control upper gastrointestinal endoscopy as a periodic screening for neoplasia. PPI therapy was stopped at least 1 week before endoscopy. Negative control group consisted of patients without GERD symptoms, not treated with PPI, with normal findings at upper gastrointestinal endoscopy. The study protocol has been approved by a local Ethics Committee (approval number R-I-002/115/2012). Patients signed informed consent before the examination. The study design is presented in Figure 1.

All patients underwent endoscopy with the use of an endoscope that allows optical magnification up to ×115 (Olympus GIF Q160Z, Olympus, Tokyo, Japan) with the distal attachment (Olympus^®^ D-201-12402, Olympus, Tokyo, Japan) for stable view and focal distance. Patients received topical oropharyngeal anesthesia by administration of 1% lidocaine. Midazolam was given intravenously according to the individual needs. Endoscopies were performed by one endoscopist experienced in magnifying endoscopy (J.W.-B.).

**Figure 2:**
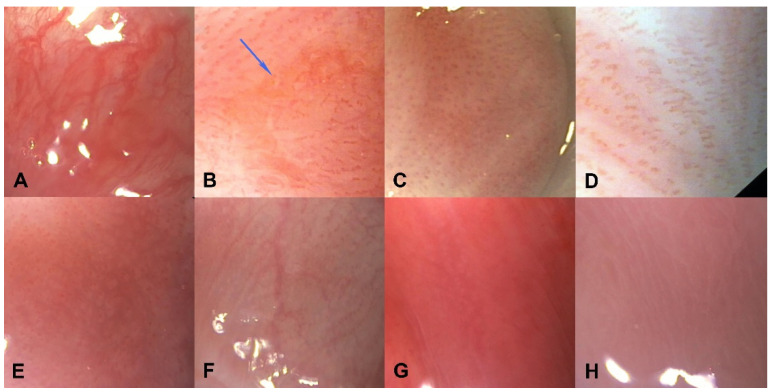
Normal squamous mucosa and minimal changes characteristic of GERD in magnifying endoscopy. (**A**)—normal squamous mucosa: palisade vessels clearly visible, no enlarged intrapapillary capillary loops (IPCLs); (**B**)—microerosion pointed by blue arrow, enlarged IPCLs in the background; (**C**)—enlarged IPCLs, palisade vessels obscured; (**D**)—elongated IPCLs; (**E**)—white spots encircling enlarged IPCLs; (**F**)—enlarged IPCLs (right side of picture) and white spots visible regardless of IPCLs (left side of the picture); (**G**)—white spots; (**H**)—palisade vessels obscured.

During conventional endoscopic evaluation, GERD patients were assigned to EE, BE, or NERD group according to the Montreal consensus guidelines [26]. Then, the squamous epithelium proximal to Z line was viewed in magnification to detect minimal lesions such as: 1. microerosions—mucosal breaks invisible prior to magnification (Figure 2B); 2. abnormal IPCLs—increased in number or enlarged (elongated, dilated) (Figure 2B–F); 3. white points—whitish spots encircling IPCLs (Figure 2E) or occurring independently from IPCLs (Figure 2F,G); and 4. obscured palisade vessels—loss of visibility of palisade vessels with the exception of the area adjacent to the Z-line, where obscured vessels may be physiological [12,13,15] (Figure 2C,E,G,H).

Next, samples for histological evaluation from squamous mucosa 1–2 cm proximal to the Z line were collected in all the groups, including BE. If any abnormalities were detected in magnifying endoscopy, biopsies were collected from the places with most apparent lesions, omitting macroscopically visible erosions. If no lesions were visible in magnification, biopsies from four quadrants of esophageal circumference were collected. After hematoxylin-eosin staining, all specimens were evaluated for the presence of typical GERD features in light microscopy: papillae elongation (feature expressed as papillae length as percent of epithelial thickness), basal cell layer hyperplasia (feature expressed as basal cell layer thickness as percent of whole epithelial thickness), and infiltrating inflammatory cells count, together making up histologic inflammation grade [5,27]. Moreover, the number of the papillae in large field of view, number of IPCLs per papilla, appearance of IPCLs (absent or normal versus enlarged), as well as the presence of DIS were assessed.

The expression of VEGF, VEGFR, EGF, and EGFR in vessels and squamous epithelium of esophagus was evaluated semi-quantitatively by immunohistochemistry. Tissue blocks were cut on a microtome into 4-µm thick sections on salinized slides. The sections were deparaffinized in xylene and hydrated in alcohols at room temperature. Then, sections were heated in a water bath for 20 min in citrate buffer (pH, 6.0) for antigen retrieval. For blocking endogenous peroxidase activity, 3% hydrogen peroxide solution was used for 10 min at room temperature. For blocking non-specific antibodies, binding horse serum was used (anti-mouse/rabbit serum produced in horse; Vector Laboratories, Inc., Burlingame, CA, USA) for 10 min at room temperature. Next, the sections were incubated with mouse anti-VEGF antibody (V4758, Sigma Aldrich/Merck, Saint Louis, MO, USA, dilution 1:20), rabbit anti-VEGFR antibody (SAB2101239, Sigma Aldrich/Merck, dilution 1:100), mouse anti-EGF antibody (SAB5300488, Sigma Aldrich/Merck, dilution 1:100), rabbit anti-EGFR antibody (HPA018530, Sigma Aldrich, Saint Louis, MO, USA, dilution 1:100), and anti-P-cadherin (Sigma Aldrich, dilution 1:100) for 60 min at room temperature. Next, the one-step system ImmPRESS™ Universal Antibody Polymer Reagent (30 min at room temperature, MP-7500, Vector Laboratories, Burlingame, CA, USA) and chromogen ImmPACT DAB (5 min at room temperature, SK-4105, Vector Laboratories) were used. Cellular nuclei were stained with hematoxylin for 5 min at room temperature. Positive and negative controls were performed according to the manufacturer’s protocol (Sigma-Aldrich/Merck).

**Figure 3:**
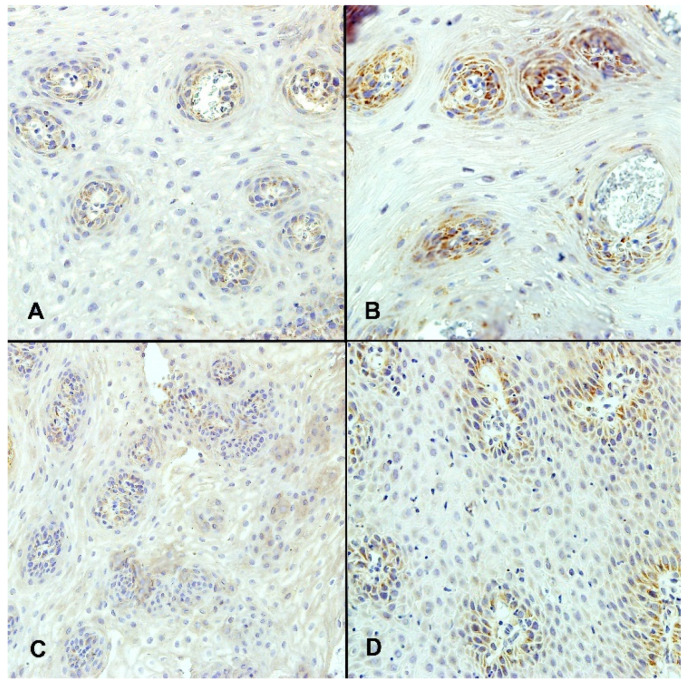
The expression of VEGF and VEGFR in esophageal squamous mucosa evaluated by immunohistochemistry. (**A**)—low VEGF expression (positive in less than 33% of vessels) (magn. ×400); (**B**)—high VEGF expression (positive in over 66% of vessels) (magn. ×400); (**C**)—low VEGFR expression (positive in less than 33% of vessels) (magn. ×200); (**D**)—high VEGFR expression (positive in over 66% of vessels) (magn. ×400).

**Figure 4:**
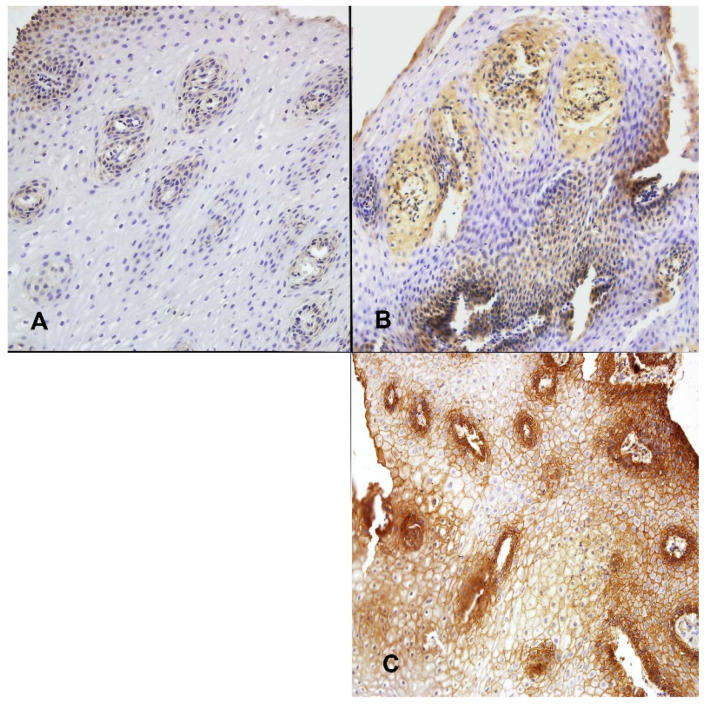
The expression of EGF and EGFR in squamous epithelium evaluated by immunohistochemistry. (**A**)—low EGF expression (positive in less than 33% of vessels and no reaction in squamous epithelium) (magn. ×200); (**B**)—high EGF expression (positive in over 66% of vessels and positive reaction in more than a half of the thickness of the squamous epithelium) (magn. ×200); (**C**)—high EGFR expression (positive in over 66% of vessels and positive reaction in more than a half of the thickness of the squamous epithelium) (magn. ×200). There were no cases with low EGFR expression in the study group.

The reaction of VEGF and VEGFR proteins was detected only in vessels of esophageal mucosa and defined as low (positive in less than 33% of vessels) (Figure 3A,C), moderate (positive in 33–66% of vessels), or high (positive in over 66% of vessels) (Figure 3B,D). The EGF and EGFR proteins expressions were analyzed in vessels and squamous epithelium and defined as low (positive in less than 33% of vessels and no reaction in squamous epithelium) (Figure 4A), moderate (positive in 33–66% of vessels and positive reaction in less than a half of the thickness of the squamous epithelium), or high (positive in over 66% of vessels and strong positive reaction in more than a half of the thickness of the squamous epithelium) (Figure 4B,C).

Pathologists examining H&E stained samples, and evaluating growth factors’ expression in immunohistochemistry were blinded to the results of conventional and magnifying endoscopy.

Statistical analysis was conducted using R software, version 4.0.5 (R Core Team (2021). R: Language and environment for statistical computing by R Foundation for Statistical Computing, Vienna, Austria, https://www.R-project.org/). Normality of distribution was assessed using Shapiro–Wilk test and was based on skewness and kurtosis values. Data for EGFR, EGF, VGFR, VGF expressions were analyzed as ordinal variables (1—low, 2—moderate, 3—high) using non-parametric tests. Comparison of groups was made with Fisher exact test for nominal variables and with *t*-test/ANOVA or their non-parametric equivalents (Mann–Whitney U test/Kruskal–Wallis test) for ordinal and continuous variables, as appropriate. Dunn post-hoc test was used, and Bonferroni correction was applied for multiple comparisons. Correlation between ordinal and continuous variables was verified with Spearman’s correlation coefficient. All the differences were considered significant at the level of *p* < 0.05.

## 3. Results

The study group consisted of a total of 50 patients: 15 patients with BE, 7 patients with EE, 20 patients with NERD, and 8 controls. There was no significant difference in age between the groups (*p* = 0.416).

The expression of VEGF and VEGFR was present only in the cytoplasm of endothelial cells. EGF and EGFR expressions were observed both in the cytoplasm of endothelial cells and in the membrane and cytoplasm of squamous epithelium cells.

**Figure 5:**
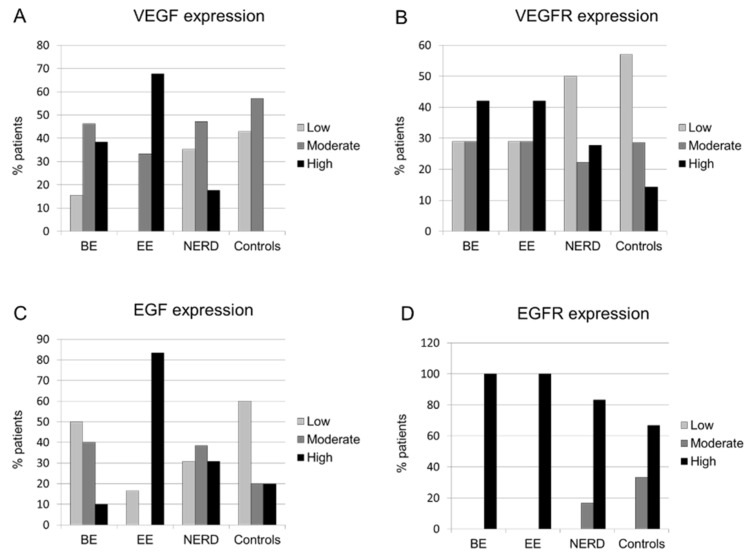
The expression of growth factors in squamous epithelium of patients with Barrett’s esophagus (BE), erosive esophagitis (EE), nonerosive reflux disease (NERD), and healthy controls. (**A**)—VEGF, (**B**)—VEGFR, (**C**)—EGF, (**D**)—EGFR. VEGF expression was significantly higher in EE than in NERD and control group. Other differences were not significant.

VEGF expression was significantly different between the groups (*p* = 0.024), with higher expression found in EE patients than NERD patients and controls. It was also higher in EE group than in BE group, but the difference was not significant (Table 1). The high expression of VEGF (positive in over 66% of vessels) was found in 15% of NERD patients, 33% of BE patients, 57% of EE patients, and none of the controls. The low VEGF expression was found in about 30% of patients with NERD and controls, in 13% of BE patients, and in none of EE patients (Figure 5).

The highest EGF expression was observed again in the EE patients, but differences with the other groups did not reach the level of statistical significance. In addition, there were no significant differences in the VEGFR and EGFR expressions between the study groups (Table 1, Figure 5).

The VEGF expression was significantly associated with presence of white spots proximal to the Z-line in study group (*p* = 0.042). Patients with white spots had significantly higher levels of VEGF expression than patients without white spots. There was no correlation between VEGF expression and the IPCLs appearance as well as the remaining endoscopic and histologic features. There was no significant correlation among the expression of VEGFR, EGF, endoscopic, and histologic features.

The expression EGFR was significantly and positively correlated with basal cell hyperplasia (*r* = 0.40, *p* = 0.045). Additionally, patients with absent or normal IPCLs on histology had lower EGFR expression than patients with elongated or widened IPCLs (*p* = 0.019). Table 2 and Table 3.

## 4. Discussion

Although GERD is one of the most common gastrointestinal problems with potentially serious complications, the pathophysiology of this disease is not fully clear. Especially, it is poorly understood why some patients develop mucosal injuries and complications and others do not. The severity of reflux measured by symptoms and pH-metric values is a poor predictor of esophageal mucosal damage and cannot satisfactorily explain this issue [1,10,11]. Therefore, pathophysiological concept of GERD is still evolving. Among the risk factors known for a long time, there are: poorly functioning antireflux barrier composed of the LES and crural diaphragm, delayed gastric emptying, number of reflux episodes, composition of refluxates coupled with impaired esophageal clearance, and alterations in esophageal mucosal integrity. The ACG guidelines for diagnosis and management of GERD issued in 2021 for the first time also enumerate release of cytokines and chemokines as a factor that might contribute to GERD symptoms and development of reflux esophagitis [6]. The number of studies concerning this issue is so far small and the results are not homogenous.

Traditionally, it has been considered that reflux esophagitis is caused by the caustic effects of refluxed gastric acid on esophageal epithelial cells. However, caustic chemical injuries develop rapidly whereas esophagitis might not appear until weeks after the induction of reflux in animal models [28]. A human study showed that after stopping PPI medication, the changes in histology such as T lymphocyte-predominant esophageal inflammation, basal cell hyperplasia, and papillary elongation appear before the loss of surface epithelial cells (erosions) [29]. An alternative concept for the development of reflux esophagitis is that refluxed gastric juice does not directly damage the esophagus, but rather stimulates esophageal epithelial cells to secrete chemokines that mediate damage of esophageal tissue [6,28,30]. Several cytokines and growth factors that play a role in tissue damage and regeneration processes may be involved in esophageal inflammation, formation of erosions, and healing. In the present study we assessed the expression of VEGF, EGF, and their receptors in patients with different GERD phenotypes and controls. We also tried to explain the role of this expression by checking how it correlates with the findings from magnifying endoscopy and histology.

VEGF is a well-known factor that stimulates cells proliferation, migration, and differentiation and regulates the formation of new vessels [18]. We found significantly higher VEGF expression in squamous mucosa of patients with erosive esophagitis compared to NERD patients or controls. The difference between EE and Barrett’s patients was not significant. The same results with regard to EE, NERD, and non-esophagitis group were presented by Taddei et al. [21]. In their study, the highest expression of VEGF was found in BE patients’ group, which can be explained by a different biopsy site (metaplastic epithelium) in Taddei et al.’s study and squamous epithelium in ours. Because in our study we concentrated on inflammatory and squamous epithelium repair processes and not on metaplastic or neoplastic transformations, we decided to biopsy squamous epithelium in all groups, including BE.

Keeping in mind the new concept that mucosal injury is rather a result than reason of the inflammation, it is very difficult to judge if high VEGF expression in EE patients found in our study can be a response to previous injuries and expression of healing or vice versa: the cause of erosions in the mechanism of increased vessels’ permeability. However, there were no significant correlations between any of the studied growth factors or receptors and presence of DIS, which reflects the increased permeability of epithelium. This fact may stand for the protective role of VEGF.

EGF is another essential cytokine in tissue damage, inflammation, and regeneration [23]. The data on the EGF expression in GERD patients are inconsistent. In one study, the levels of EGF were decreased in esophageal papillae and capillary endothelium in GERD patients [25]. In another, EGF expression was higher in the area of reflux esophagitis than in the proximally located areas with normal epithelium [20]. In our study, the highest EGF expression was observed in the EE patients, but differences with the other groups did not reach the level of statistical significance, possibly due to small sizes of the groups. In addition, there were no significant differences in the EGFR expressions between the study groups.

Pretto et al. found that the frequency of positive EGFR immunohistostaining increased from patients with GERD, through BE to esophageal adenocarcinoma [31]. There were no division for NERD and EE in the GERD group in their study and the biopsies from BE patients were collected from metaplastic segment. In the rat model, Fujiwara et al. showed that EGFR expression was significantly increased in a chronic reflux esophagitis compared with normal esophageal mucosa [32]. The exogenous administration of EGF prevented the increased severity of esophagitis in rats after sialoadenectomy. They also proved on human esophageal cell lines that EGF had a cytoprotective effect against acid-induced cell injury.

It has previously been found that luminal release of EGF was significantly lower in patients with EE compared with controls during perfusion with HCl and this was considered as one of the potential mechanisms leading to damage of the esophageal mucosa during gastroesophageal reflux episodes [24]. Apart from the epithelium itself, esophageal mucosal glands and swallowed saliva may be an important source of EGF in esophagus. Salivary secretion of EGF was stronger in NERD patients and weaker in EE patients in comparison to asymptomatic controls [24,33]. This enhanced salivary esophagoprotection can potentially mediate resistance to the development of mucosal changes in GERD. In the study of Tobey et al., the change in shunt permeability enabled luminal EGF to diffuse across the acid-damaged epithelium and through this to access its receptors on epithelial basal cells [34]. The authors hypothesized that the shunt leak of EGF may, in part, account for the development of a reparative phenomenon known as basal cell hyperplasia. In our study, the EGFR expression correlated positively with basal cell hyperplasia and patients with absent or normal IPCLs in histology had lower EGFR expression than patients with elongated or widened IPCLs.

A significant increase in the density of blood vessels is commonly observed during the proliferative phase of healing [19]. This may reflect an increase of the number and size of IPCLs visible in magnifying endoscopy in GERD patients. A previously published study described vessels abnormalities in esophageal squamous mucosa qualified as minimal endoscopic changes accompanying GERD [12,13,14,15]. The authors found, among others, obscured palisade vessels, more numerous, elongated, and widened IPCLs, and white spots that surrounded IPCLs or existed independently from them. The presence of white points correlated positively with increased number and length of papillae, as well as with inflammatory cells infiltration and acantosis. Moreover, the arrangement of white points was similar to the distribution of pathological IPCLs [13]. In the present study, among all endoscopic minimal changes, the presence of white spots correlated with increased expression of VEGF. Interestingly, we did not find correlation between abnormal (numerous, elongated, or widened) IPCLs and VEGF expression. This observation may be explained by the fact that the expression of VEGF is no longer maintained in such fully developed vessels. So far, it is not known which histologic structure or other phenomenon is responsible for the presence of white spots. Taking into account the finding that the VEGF expression is a strong stimulator of angiogenesis and was significantly higher in the areas presenting white spots in magnification, together with the usual distribution of white spots, we hypothesize that white spots are the precursors of IPCLs or elongated mucosal papillae. Explaining the substrate for white points may allow the assignment of them to the already known histopathological features of esophagitis. If future studies prove that white points are elongated papillae (the structures that appear early after discontinuation of PPI [29]), that will improve the diagnostic accuracy of endoscopy for GERD in patients previously treated with PPI. This finding, after some future studies on its accuracy, may contribute to the reduction of the need for biopsy.

The change in the paradigm of mucosal injuries pathogenesis in GERD from a simple chemical acid injury into cytokine-dependent sequel opens up new treatment options. PPI, which are nowadays a mainstay of GERD treatment, do not cause satisfactory symptom relief in up to 32–45% of patients [35]. Moreover, there is a subgroup of patients that despite chronic treatment with PPI have non healing esophageal ulcers leading to stenosis and require repetitive endoscopic esophageal dilatation. These groups would possibly benefit from some anti-inflammatory treatment, perhaps cytokine or growth factors influencing drags analogically to patients with inflammatory bowel diseases receiving biological treatment. However, before such treatment may be searched, we must better understand the pathophysiology of esophageal inflammation.

As the VEGF expression is not higher in NERD than in EE patients, it is not responsible for preventing erosions but it is released as a response to injury. Correlation with white points instead of abnormal (elongated and numerous) IPCLs may mean that VEGF in GERD is important, mainly in an early stage of healing. Lack of correlation between VEGF expression and DIS is the argument that it does not cause detrimental excessive permeability of epithelium. EGFR expression correlates positively with basal cell hyperplasia. Patients with enlarged IPCLs in histology have higher EGFR expression than patients with absent or normal IPCLs, which may speak for its role in vessel creation. These considerations emerging from our study should be considered as hypotheses for further testing.

Because of single center execution of our study and small number of cases enrolled, the results should be treated as preliminary and require confirmation on larger groups, possibly including patients with GERD complicated with esophageal stenosis.

To our knowledge, this is the first study describing a relationship between the minimal endoscopic lesions in GERD and growth factors expression. Our approach, which combines the assessment of growth factors with the findings of magnifying endoscopy and histopathology, provides a good insight in the pathogenesis of mucosal injury and repair in GERD patients, so in our opinion is worth following. It may also help to understand the origin and character of minimal endoscopic lesions and bridge the clinical with molecular findings. This is part of the current trend of endoscopic search for endoscopic NERD markers.

## 5. Conclusions

In conclusion, patients with erosive esophagitis have significantly increased expression of VEGF but not EGF or their receptors than NERD patients and healthy people. VEGF may play a role in the formation of white spots as its expression positively correlates with the presence of white spots on endoscopy. Based on these findings, as well as the results of previous studies, we hypothesize that white spots may be the precursors of IPCLs or elongated mucosal papillae. In addition, the EGFR expression correlates with basal cell hyperplasia and enlarged IPCLs in histology.

## Figures and Tables

**Table 1:** Age and expression of VEGF, VEGFR, EGF, and EGFR in the study groups.

	BE (*n* = 15)	EE (*n* = 7)	NERD (*n* = 20)	Controls (*n* = 8)	Test Statistics	Effect Size [95% CI]	*p*	Post-Hoc Test
Age, years, mean ± SD	54.50 ± 15.47	47.14 ± 26.35	44.75 ± 16.58	52.86 ± 19.89	F = 0.673 df = 1; 46	η = 0.01 [0.00; 0.12]	0.4160	
VEGF, median (Q1; Q3)	2 (2; 3)	3 (2.25; 3)	2 (1; 2)	2 (1; 2)	χ^2^ = 9.459 df = 3	ε^2^ = 0.193 [0.084; 0.413]	0.0238	EE > NERD, Controls
VEGFR, median (Q1; Q3)	2 (1.25; 3)	2 (1.5; 3)	1.5 (1; 2.75)	1 (1; 2)	χ^2^ = 3.028 df = 3	ε^2^ = 0.062 [0.011; 0.295]	0.3873	
EGF, median (Q1; Q3)	1.5 (1; 2)	3 (3; 3)	2 (1; 3)	1 (1; 2)	χ^2^ = 6.644 df = 3	ε^2^ = 0.136 [0.035; 0.362]	0.0841	
EGFR, median (Q1; Q3)	3 (3; 3)	3 (3; 3)	3 (3; 3)	3 (2.25; 3)	χ^2^ = 4.950 df = 3	ε^2^ = 0.101 [0.037; 0.379]	0.1755	

Data for EGFR, EGF, VEGFR, VEGF presented as ordinal variables (1—low, 2—moderate, 3—high) with median (1st quartile—Q1; 3rd quartile—Q3). Groups compared with ANOVA (age) and with Kruskal–Wallis test with Dunn post-hoc test for remaining variables (Bonferroni correction was applied for multiple comparisons). VEGF—vascular endothelial growth factor, VEGFR—vascular endothelial growth factor receptor, EGF—epidermal growth factor, EGFR—epidermal growth factor receptor, BE—Barrett’s esophagus, EE—erosive esophagitis, NERD—non-erosive gastroesophageal reflux disease, *n*—number of patients, F—ANOVA statistics, df—degrees of freedom, χ^2^—Kruskal–Wallis statistics, ε^2^—epsilon squared effect size, η—eta effect size.

**Table 2:** Comparison of VEGF, VEGFR, EGF, and EGFR expressions against qualitative endoscopic and histologic features.

	VEGF		VEGFR		EGF		EGFR	
	Median (Q1; Q3)	*p*	Median (Q1; Q3)	*p*	Median (Q1; Q3)	*p*	Median (Q1; Q3)	*p*
Microerosions								
Present	2 (2; 3)	0.535	2 (1; 3)	0.827	2 (1; 3)	0.162	3 (3; 3)	0.707
Absent	2 (1; 3)		2 (1; 3)		2 (1; 2)		3 (3; 3)	
Palisade vessels								
Visible	2 (2; 3)	0.496	2 (1; 3)	0.913	2 (1; 3)	0.676	3 (3; 3)	0.876
Obscurred	2 (1; 3)		2 (1; 3)		2 (2; 3)		3 (3; 3)	
White spots								
Present	2.5 (2; 3)	0.042	2 (1; 3)	0.397	2.5 (2; 3)	0.157	3 (3; 3)	0.145
Absent	2 (1; 3)		1.5 (1; 3)		2 (1; 2)		3 (3; 3)	
IPCLs—endoscopy								
Abnormal	2 (1.5; 3)	0.438	2 (1; 3)	0.627	2 (1; 3)	0.355	3 (3; 3)	0.308
Normal	2 (2; 3)		2.5 (1; 3)		2 (1; 2)		3 (3; 3)	
IPCLs—histology								
Absent or normal	2 (2; 2)	0.742	2 (1; 3)	0.934	2 (1; 2)	0.499	3 (2; 3)	0.005
Enlarged	2 (1; 3)		2 (1; 3)		2 (1; 3)		3 (3; 3)	
DIS								
Absent	2 (2; 2.5)	0.815	2 (1; 3)	0.253	2 (1; 2)	0.246	3 (3; 3)	0.116
Present	2 (1,5; 3)		1.5 (1; 3)		2.5 (1; 3)		3 (3; 3)	

VEGF—vascular endothelial growth factor, VEGFR—vascular endothelial growth factor receptor, EGF—epidermal growth factor, EGFR—epidermal growth factor receptor, IPCLs—intrapapillary capillary loops, DIS—dilated intracellular spaces.

**Table 3:** Correlation of VEGF, VEGFR, EGF, and EGFR expressions with quantitative endoscopic and histologic features.

Correlation	VEGF		VEGFR		EGF		EGFR	
	*r*	*p*	*r*	*p*	*r*	*p*	*r*	*p*
Age	−0.20	0.2509	−0.04	0.7996	−0.31	0.1012	0.26	0.1854
Histologic inflammation grade	0.04	0.8035	−0.24	0.1396	0.23	0.2376	0.24	0.2196
Basal cell hyperplasia	0.01	0.9558	−0.21	0.2153	0.27	0.1887	0.40	0.0454
Number of papillae in the field of view	0.29	0.1143	−0.05	0.7697	0.07	0.7321	0.36	0.0799
Papillae length	0.28	0.1267	−0.12	0.4814	0.26	0.2068	0.26	0.2099
Number of IPCLs per papilla	0.29	0.1125	0.04	0.8091	0.01	0.9419	0.38	0.0603
Inflammatory cell count	0.20	0.2746	−0.22	0.2021	0.39	0.0570	0.01	>0.9999

*r*—Spearman’s correlation coefficient, *p*—*p*-value, VEGF—vascular endothelial growth factor, VEGFR—vascular endothelial growth factor receptor, EGF—epidermal growth factor, EGFR—epidermal growth factor receptor, IPCLs—intrapapillary capillary loops.

## Data Availability

The data supporting reported results can be obtained from the corresponding author on request.

## References

[1] Ronkainen J., Aro P., Storskrubb T., Johansson S.E., Lind T., Bolling-Sternevald E., Graffner H., Vieth M., Stolte M., Engstrand L. (2005). High prevalence of gastroesophageal reflux symptoms and esophagitis with or without symptoms in the general adult Swedish population: A Kalixanda study report. Scand. J. Gastroenterol..

[2] Dent J., El-Serag H.B., Wallander M.A., Johansson S. (2005). Epidemiology of gastro-oesophageal reflux disease: A systematic review. Gut.

[3] Jung H.K., Tae C.H., Song K.H., Kang S.J., Park J.K., Gong E.J., Shin J.E., Lim H.C., Lee S.K., Jung D.H. (2021). 2020 Seoul Consensus on the Diagnosis and Management of Gastroesophageal Reflux Disease. J. Neurogastroenterol. Motil..

[4] Peery A.F., Crockett S.D., Barritt A.S., Dellon E.S., Eluri S., Gangarosa L.M., Jensen E.T., Lund J.L., Pasricha S., Runge T. (2015). Burden of Gastrointestinal, Liver, and Pancreatic Diseases in the United States. Gastroenterology.

[5] Mastracci L., Grillo F., Parente P., Unti E., Battista S., Spaggiari P., Campora M., Scaglione G., Fassan M., Fiocca R. (2020). Gastro-esophageal reflux disease and Barrett’s esophagus: An overview with an histologic diagnostic approach. Pathologica.

[6] Katz P.O., Dunbar K.B., Schnoll-Sussman F.H., Greer K.B., Yadlapati R., Spechler S.J. (2022). ACG Clinical Guideline for the Diagnosis and Management of Gastroesophageal Reflux Disease. Am. J. Gastroenterol..

[7] Hunt R., Armstrong D., Katelaris P., Afihene M., Bane A., Bhatia S., Chen M.H., Choi M.G., Melo A.C., Fock K.M. (2017). World Gastroenterology Organisation Global Guidelines: GERD Global Perspective on Gastroesophageal Reflux Disease. J. Clin. Gastroenterol..

[8] Schneider J.L., Corley D.A. (2017). The Troublesome Epidemiology of Barrett’s Esophagus and Esophageal Adenocarcinoma. Gastrointest. Endosc. Clin. N. Am..

[9] Grillo F., Mastracci L., Saragoni L., Vanoli A., Limarzi F., Gullo I., Ferro J., Paudice M., Parente P., Fassan M. (2020). Neoplastic and pre-neoplastic lesions of the oesophagus and gastro-oesophageal junction. Pathologica.

[10] Grande M., Sileri P., Attinà G.M., De Luca E., Ciano P., Ciangola C.I., Cadeddu F. (2012). Nonerosive gastroesophageal reflux disease and mild degree of esophagitis: Comparison of symptoms endoscopic, manometric and pH-metric patterns. World J. Surg. Oncol..

[11] Dent J., Becher A., Sung J., Zou D., Agréus L., Bazzoli F. (2012). Systematic review: Patterns of reflux-induced symptoms and esophageal endoscopic findings in large-scale surveys. Clin. Gastroenterol. Hepatol..

[12] Edebo A., Tam W., Bruno M., Van Berkel A.M., Jönson C., Schoeman M., Tytgat G., Dent J., Lundell L. (2007). Magnification endoscopy for diagnosis of nonerosive reflux disease: A proposal of diagnostic criteria and critical analysis of observer variability. Endoscopy.

[13] Wasielica-Berger J., Kemona A., Kiśluk J., Świdnicka-Siergiejko A., Rogalski P., Chwieśko A., Kostrzewska M., Dąbrowski A. (2018). The added value of magnifying endoscopy in diagnosing patients with certain gastroesophageal reflux disease. Adv. Med. Sci..

[14] Kiesslich R., Kanzler S., Vieth M., Moehler M., Neidig J., Thanka Nadar B.J., Schilling D., Burg J., Nafe B., Neurath M.F. (2004). Minimal change esophagitis: Prospective comparison of endoscopic and histological markers between patients with non-erosive reflux disease and normal controls using magnifying endoscopy. Dig. Dis..

[15] Netinatsunton N., Sottisuporn J., Attasaranya S., Witeerungrot T., Chamroonkul N., Jongboonyanuparp T., Geater A., Ovartlarnporn B. (2016). i-Scan detection of minimal change esophagitis in dyspeptic patients with or without Gastroesophageal Reflux disease. BMC Gastroenterol..

[16] Mastracci L., Grillo F., Parente P., Unti E., Battista S., Spaggiari P., Campora M., Valle L., Fassan M., Fiocca R. (2020). Non gastro-esophageal reflux disease related esophagitis: An overview with a histologic diagnostic approach. Pathologica.

[17] Savarino E., Zentilin P., Mastracci L., Dulbecco P., Marabotto E., Gemignani L., Bruzzone L., de Bortoli N., Frigo A.C., Fiocca R. (2013). Microscopic esophagitis distinguishes patients with non-erosive reflux disease from those with functional heartburn. J. Gastroenterol..

[18] Johnson K.E., Wilgus T.A. (2014). Vascular Endothelial Growth Factor and Angiogenesis in the Regulation of Cutaneous Wound Repair. Adv. Wound Care.

[19] Brown N.J., Smyth E.A., Cross S.S., Reed M.W. (2002). Angiogenesis induction and regression in human surgical wounds. Wound Repair. Regen..

[20] Luo J.C., Lin H.Y., Lu C.L., Chen T.S., Lin H.C., Li C.P., Liao W.C., Chang F.Y., Lee S.D. (2008). Growth factors expression in patients with erosive esophagitis. Transl. Res..

[21] Taddei A., Fabbroni V., Pini A., Lucarini L., Ringressi M.N., Fantappiè O., Bani D., Messerini L., Masini E., Bechi P. (2014). Cyclooxygenase-2 and inflammation mediators have a crucial role in reflux-related esophageal histological changes and Barrett’s esophagus. Dig. Dis. Sci..

[22] Inamori M., Shimamura T., Nagase H., Abe Y., Umezawa T., Nakajima A., Saito T., Ueno N., Tanaka K., Sekihara H. (2006). mRNA expression of inducible nitric oxide synthase, endothelial nitric oxide synthase and vascular endothelial growth factor in esophageal mucosa biopsy specimens from patients with reflux esophagitis. Hepatogastroenterology.

[23] Bodnar R.J. (2013). Epidermal Growth Factor and Epidermal Growth Factor Receptor: The Yin and Yang in the Treatment of Cutaneous Wounds and Cancer. Adv. Wound Care.

[24] Rourk R.M., Namiot Z., Edmunds M.C., Sarosiek J., Yu Z., McCallum R.W. (1994). Diminished luminal release of esophageal epidermal growth factor in patients with reflux esophagitis. Am. J. Gastroenterol..

[25] Jankowski J., Coghill G., Tregaskis B., Hopwood D., Wormsley K.G. (1992). Epidermal growth factor in the oesophagus. Gut.

[26] Vakil N., van Zanten S.V., Kahrilas P., Dent J., Jones R., Global Consensus Group (2006). The Montreal definition and classification of gastroesophageal reflux disease: A global evidence-based consensus. Am. J. Gastroenterol..

[27] Vieth M., Peitz U., Labenz J., Kulig M., Nauclér E., Jaspersen D., Meyer-Sabellek W., Willich S., Lind T., Malfertheiner P. (2004). What parameters are relevant for the histological diagnosis of gastroesophageal reflux disease without Barrett’s mucosa?. Dig. Dis..

[28] Souza R.F., Huo X., Mittal V., Schuler C.M., Carmack S.W., Zhang H.Y., Zhang X., Yu C., Hormi-Carver K., Genta R.M. (2009). Gastroesophageal reflux might cause esophagitis through a cytokine-mediated mechanism rather than caustic acid injury. Gastroenterology.

[29] Dunbar K.B., Agoston A.T., Odze R.D., Huo X., Pham T.H., Cipher D.J., Castell D.O., Genta R.M., Souza R.F., Spechler S.J. (2016). Association of Acute Gastroesophageal Reflux Disease With Esophageal Histologic Changes. JAMA.

[30] Souza R.F., Bayeh L., Spechler S.J., Tambar U.K., Bruick R.K. (2017). A new paradigm for GERD pathogenesis. Not acid injury, but cytokine-mediated inflammation driven by HIF-2α: A potential role for targeting HIF-2α to prevent and treat reflux esophagitis. Curr. Opin. Pharmacol..

[31] Pretto G., Gurski R.R., Binato M., Navarini D., Aguiar W.W., Meurer L. (2013). Increase of epidermal growth factor receptor expression in progression of GERD, Barrett, and adenocarcinoma of esophagus. Dig. Dis. Sci..

[32] Fujiwara Y., Higuchi K., Takashima T., Hamaguchi M., Hayakawa T., Tominaga K., Watanabe T., Oshitani N., Shimada Y., Arakawa T. (2006). Roles of epidermal growth factor and Na^+^/H^+^ exchanger-1 in esophageal epithelial defense against acid-induced injury. Am. J. Physiol. Gastrointest. Liver Physiol..

[33] Yandrapu H., Marcinkiewicz M., Poplawski C., Han K., Zbroch T., Goldin G., Sarosiek I., Namiot Z., Sarosiek J. (2015). Role of saliva in esophageal defense: Implications in patients with nonerosive reflux disease. Am. J. Med. Sci..

[34] Tobey N.A., Hosseini S.S., Argote C.M., Dobrucali A.M., Awayda M.S., Orlando R.C. (2004). Dilated intercellular spaces and shunt permeability in nonerosive acid-damaged esophageal epithelium. Off. J. Am. Coll. Gastroenterol..

[35] El-Serag H., Becher A., Jones R. (2010). Systematic review: Persistent reflux symptoms on proton pump inhibitor therapy in primary care and community studies. Aliment. Pharmacol. Ther..

